# The Stinging Tree

**Published:** 1882-11

**Authors:** 


### THE STINGING TREE.

The "stinging tree" of Queensland is a luxurious shrub, pleasing to the eye, but dangerous to the touch. It grows from two or three inches to ten or fifteen feet in height, and emits a disagreeable odor. Says a traveler :

Sometimes while shooting turkeys in the scrubs I have entirely forgotten the stinging tree, till I was warned of its close proximity by its smell, and have often found myself in a little forest of them. I was only once stung, and that very lightly.

Its effects are curious ; it leaves no mark, but the pain is maddening ; and for months afterward the part when touched is tender in rainy weather, or when it gets wet in washing, etc. I have seen a man who treats ordinary pain lightly roll on the ground in agony after being stung, and I have known a horse so completely mad after getting into a grove of the trees that he rushed open-mouthed at every one who approached him, and had to be shot. Dogs, when stung, will rush about, whining piteously, biting pieces from the affected part.

